# Troglitazone and Δ2Troglitazone Enhance Adiponectin Expression in Monocytes/Macrophages through the AMP-Activated Protein Kinase Pathway

**DOI:** 10.1155/2014/726068

**Published:** 2014-09-22

**Authors:** Jaw-Shiun Tsai, Lee-Ming Chuang, Ching-Shih Chen, Chan-Jung Liang, Yuh-Lien Chen, Ching-Yu Chen

**Affiliations:** ^1^Department of Family Medicine, College of Medicine, National Taiwan University, Taipei 10051, Taiwan; ^2^Department of Internal Medicine, College of Medicine, National Taiwan University, Taipei 10051, Taiwan; ^3^Division of Medicinal Chemistry, College of Pharmacy and Comprehensive Cancer Center, The Ohio State University, Columbus, OH 43210, USA; ^4^Institute of Biological Chemistry, Academia Sinica, Taipei 11529, Taiwan; ^5^Department of Anatomy and Cell Biology, College of Medicine, National Taiwan University, Taipei 10051, Taiwan; ^6^Division of Geriatric Research, Institute of Population Health Science, National Health Research Institutes, Miaoli 35053, Taiwan

## Abstract

Accumulating evidence indicates that the regimen to increase adiponectin will provide a novel therapeutic strategy for inflammation and cardiovascular disorders. Here, we tested the effect of troglitazone (TG) and its newly synthesized derivative, 5-[4-(6-hydroxy-2,5,7,8-tetramethyl-chroman-2-yl-methoxy)-benzylidene]-2,4-thiazolidinedione (Δ2troglitazone, (Δ2TG)), on the adiponectin expression in monocytes/macrophages and the relative mechanisms. The expression of adiponectin was located in macrophages of atherosclerotic lesions from patients and cholesterol-fed rabbits. TG and Δ2TG enhanced adiponectin mRNA and protein expression in THP-1 cells by quantitative real-time PCR, Western blot, and immunocytochemistry. TG induced adiponectin mRNA expression through a PPAR*γ*-dependent pathway whereas Δ2TG enhanced adiponectin mRNA expression through a PPAR*γ*-independent pathway in THP-1 cells. Both TG and Δ2TG enhanced adiponectin mRNA expression through AMP-activated protein kinase (AMPK) activation. TG and Δ2TG decreased the adhesion of THP-1 cells to TNF-α-treated HUVECs and the inhibitory effect was abolished by specific antiadiponectin antibodies. TG- and Δ2TG-induced suppression on monocyte adhesion were inhibited by a selective AMPK inhibitor compound C. Our data suggest that the inhibitory effect of TG and Δ2TG on monocyte adhesion might be at least in part through *de novo* adiponectin expression and activation of an AMPK-dependent pathway, which might play an important role in anti-inflammation and antiatherosclerosis.

## 1. Introduction

Macrophages are heterogenous and plastic population of phagocytic cells, which arise from circulating myeloid-derived blood monocytes, enter target tissues, and gain phenotypic and functional attributes partly determined by their tissue of residence [1]. These cells play a crucial role in the processes of inflammation and cardiovascular disorders. They accumulate large amounts of lipid to form the foam cells that initiate the formation of the lesion and participate actively in the development of the atherosclerotic lesion. A well-characterized cell model system to study this critical transformation of macrophages to foam cells is the human THP-1 monocytic cell line [2]. Adiponectin, an adipocytokine exclusively expressed and secreted by adipocytes and circulating in plasma in a high concentration, has been shown to inhibit macrophage foam cell formation by downregulating scavenger receptor A expression and acyl-coenzyme A: cholesterol acyltransferase-1 expression [3]. Although adiponectin has been considered to be expressed and secreted largely from the adipose tissue, adiponectin mRNA expression has been found in several other cell types, including primary hepatic sinusoidal endothelial cells, stellate cells, and macrophages [4]. It has also been reported that adiponectin may inhibit both the inflammatory process and atherogenesis by suppressing the migration of monocytes/macrophages, the transformation into macrophage foam cells, and the lipid accumulation in macrophages [5, 6]. Thus, the increasing adiponectin expression has become a promising drug target for the treatment of cardiovascular and other related disorders.

**Figure 1:**
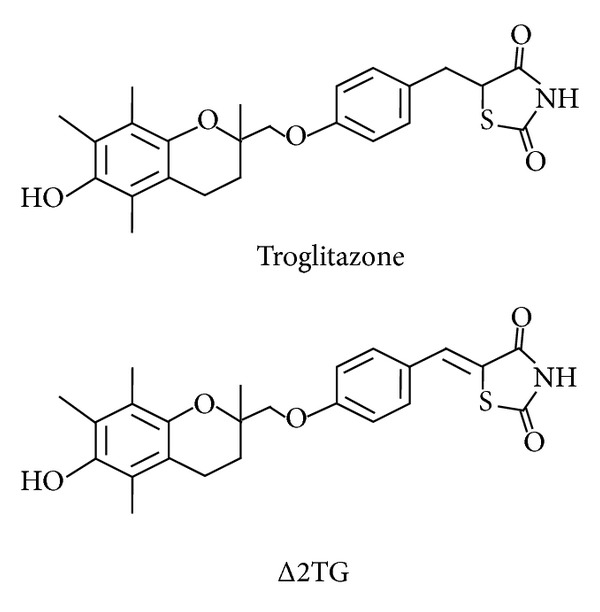
Chemical structures of troglitazone and its PPAR*γ*-inactive analogues Δ2troglitazone (Δ2TG). The introduction of the double bond adjoining the terminal thiazolidinedione ring results in the abrogation of the PPAR*γ* ligand property of Δ2TG.

The thiazolidinediones have emerged as effective agents for antidiabetes and anti-inflammation [7]. It is generally assumed that they function by activating peroxisome proliferator-activated receptor-*γ* (PPAR*γ*). The thiazolidinediones-induced adiponectin expression through PPAR*γ* activation in adipocytes may underlie its pharmacological functions, as adiponectin contributing to insulin-sensitizing and antiatherogenic effects is well established [8]. Troglitazone, a PPAR*γ* activator, reduced tumor necrosis factor-alpha (TNF)-α-induced reactive oxygen species (ROS) production and intercellular adhesion molecule-1 (ICAM-1) expression in endothelial cells [9]. PPAR*γ* activators enhance the expression of PPAR*γ* in macrophages and inhibit synthesis of scavenger receptor A and matrix metalloproteinase-9 [10]. Our previous study demonstrated that PPAR*γ* agonist rosiglitazone inhibits monocyte adhesion to fibronectin-coated plates through* de novo* adiponectin production in human monocytes [11]. The function of thiazolidinediones may improve insulin sensitivity by increasing concentrations of adiponectin and by decreasing free fatty acid and inflammatory factor TNF-α levels in diabetic subjects and animal models [12, 13]. Regulation of adiponectin expression requires a complex array of intracellular signaling pathways involving PPAR*γ* and AMPK [14, 15]. Little is known about the effects of troglitazone (TG) and its newly synthesized derivative, 5-[4-(6-hydroxy-2,5,7,8-tetramethyl-chroman-2-yl-methoxy)-benzylidene]-2,4-thiazolidinedione (Δ2troglitazone (Δ2TG), Figure 1) on adiponectin expression under inflammatory conditions and the mechanisms of these effects, and a better understanding of these points might provide important insights into the development of inflammation and cardiovascular disorders. The aims of this study were to investigate the effects of TG and Δ2TG on the adiponectin expression in THP-1 cells and to determine whether PPAR*γ* and AMPK were involved. Our results showed that TG and Δ2TG increased adiponectin mRNA and protein expression and that this effect was mediated by AMPK phosphorylation. TG and Δ2TG also significantly reduced the adhesion of the monocytes to TNF-α-treated HUVECs.

## 2. Materials and Methods

### 2.1. Sample Collection and Immunohistochemical Staining

This study was approved by the Institutional Review Board of the National Taiwan University Hospital, Taipei, Taiwan. All participants provided written informed consent before inclusion in the study. All experimental procedures and protocols involving animals were in accordance with the local institutional guidelines for animal care, were approved by the Institutional Animal Care Committee of the National Taiwan University (Taipei, Taiwan), and complied with the Guide for the Care and Use of Laboratory Animals (NIH publication no. 86-23, revised 1985). Coronary arteries were obtained from 3 patients undergoing surgery for cardiac transplantation or atherosclerosis. Immediately after surgery, tissues were rinsed with ice-cold phosphate-buffered saline (PBS), fixed in 4% paraformaldehyde solution, and paraffin-embedded. Tissues were serially sectioned at 5 *μ*m intervals and the tissue sections were deparaffinized, rehydrated, and washed with PBS. Endogenous peroxidase activity was eliminated by incubation with 3% H_2_O_2_. Sections were then incubated with PBS containing 5 mg/mL bovine serum albumin (BSA) to block nonspecific binding.

To determine the level of adiponectin expression in vascular walls and whether it was associated with macrophages, two serial sections were examined by immunostaining for, respectively, adiponectin or a marker for macrophages. The first section was incubated sequentially for overnight at 4°C with a 1 : 100 dilution of rabbit antibodies against human adiponectin (Epitomics) in phosphate-buffered saline (PBS) containing 10% normal horse serum (Gibco) (PBS-NHS) and for 90 min at room temperature with a 1 : 200 dilution of biotinylated goat anti-rabbit IgG antibodies (Santa Cruz Biotechnology) in PBS-NHS, then bound antibodies were visualized using 3,3′-diaminobenzidine (DAB, Sigma-Aldrich). Specific signals recognized by the primary antibody are brown. As a negative control, the primary antiserum was replaced by normal rabbit immunoglobulins. For the identification of macrophages, the second section was incubated with mouse monoclonal antibodies against human macrophage (DAKO, Japan). These sections were then incubated with fluorescein isothiocyanate (FITC)-conjugated goat anti-mouse secondary antibody (Sigma) and observed by fluorescence microscopy.

### 2.2. Cell Culture

Human monocytic leukemia THP-1 cells were cultured in RPMI 1640 medium (Gibco, Life Technologies, NY, USA) supplemented with 10% fetal bovine serum, penicillin (100 U/mL, Biologival Industries, Israel), and streptomycin (100 mg/mL) at 37°C in 5% CO_2_. All reagents were added to the culture medium in a minimal volume (<0.1%) of dimethyl sulfoxide (DMSO), and in each case the carrier was shown to not affect the measured parameters. For each experiment, a minimum of three independent experiments with the triplicate samples was performed. 

### 2.3. Preparation of Cell Lysates and Western Blot Analysis

To prepare cell lysates, the cells were lysed for 1 h at 4°C in 20 mM Tris-HCl, 150 mM NaCl, 1 mM EDTA, 1 mM EGTA, 1% Triton X-100, 1 mM phenylmethylsulfonyl fluoride, and pH 7.4; then the lysate was centrifuged at 4000 g for 30 min at 4°C and the supernatant retained. Samples of cell lysate (80 *μ*g of protein) were subjected to 10% sodium dodecyl sulfate (SDS)-polyacrylamide gel electrophoresis and transferred to polyvinylidene fluoride membranes (Pall Corporation, NY, USA), which were then incubated for 30 min at room temperature with 5% nonfat milk in Tris-buffered saline containing 0.2% Tween 20 (TBST) to block nonspecific binding of antibodies. All dilutions of antibodies used were in TBST. The membranes were then incubated overnight at 4°C with rabbit antibodies against human adiponectin (Abcam; 1 : 2000 dilution) or human phospho-AMPK (Cell Signaling; 1 : 1000 dilution), then for 1 h at room temperature with horseradish peroxidase-conjugated goat anti-rabbit IgG antibodies (Sigma; 1 : 5000 dilution), bound antibodies being detected using chemiluminescence reagent Plus (NEN, Boston, MA, USA) and the intensity of each band quantified using a densitometer. Antibodies against AMPK (Cell Signaling; 1 : 1000 dilution) or *β*-actin (santa Cruz; 1 : 10000 dilution) were used as loading controls.

### 2.4. Quantitative Real-Time PCR Analysis

Total RNA was extracted by REzol (PROtech Technology, Sparks, NV), according to the manufacturer's instructions. Single-stranded cDNA was synthesized with SuperScript II reverse transcriptase (Invitrogen, Carlsbad, CA). The Q-PCR was performed with ABI 7000 real-time PCR system, with primers for measuring adiponectin (forward: 5′-AGA AAG GAG ATC CAG GTC TTA TTG GT-3′, reverse: 5′-AAC GTA AGT CTC CAA TCC CAC ACT-3′). Real-time PCR was performed with an initial denaturation at 94°C for 5 min, followed by denaturing at 94°C for 30 s, annealing at 62°C for 30 s, and polymerization at 72°C for 30 s for a total of 35 cycles, then by a final extension at 72°C for 10 min. The expression levels of mRNA were normalized by the expression of the housekeeping gene glyceraldehyde dehydrogenase (GAPDH). 

### 2.5. Immunocytochemistry

To localize adiponectin expression in situ, cells (control or cells treated for 24 h with TG or with Δ2TG) adhered to fibronectin-coated cover glasses were fixed with 4% paraformaldehyde in PBS for 15 min. After treatment with 0.1% Triton X-100 for 1 min, they were treated with bovine serum albumin in PBS (5 mg/mL) for 1 h to block nonspecific binding. The cells were incubated with adiponectin (1 : 50 dilution; R&D Systems) antibody for overnight at 4°C. They were then incubated with FITC-conjugated secondary antibodies (1 : 100 dilutions; Sigma) for 1 h at room temperature and stained with DAPI (1 : 6,000 dilutions) for 10 min. The cells were then observed by confocal fluorescent microscopy (EZ-C1; Nikon, Tokyo, Japan). Negative control was performed by omitting the incubation of the cells with primary antibodies.

### 2.6. Monocyte-Endothelial Cell Adhesion Assay

Monocytes were suspended at the concentration of 4 × 10^5^ cells per well and were cultured in serum-free medium with or without TG or Δ2TG (9 *µ*M) for 18 h. To assess the effects of adiponectin on monocyte adhesiveness to endothelial cells, THP-1 cells were preincubated for 30 min with adiponectin antibody (Abcam, UK) or with GW9662 or with an AMP-dependent protein kinase (AMPK) inhibitor compound C (Merck). Subsequently the THP-1 cells were labeled for 1 h at 37°C with 1 mM BCECF/AM (Boehringer Mannheim, Mannheim, Germany) in DMSO and then were suspended in the same medium used for culture of HUVECs. Primary cultures of HUVECs were prepared as described previously [16]. The cells were grown in medium 199 (Gibco, NY, USA) containing 1% penicillin-streptomycin, 30 *μ*g/mL of endothelial cell growth supplement (R&D Systems, Minneapolis, MN), and 10% fetal bovine serum (FBS; Biological Industries, Israel) at 37°C in a humidified atmosphere of 95% air, 5% CO_2_. Cells between passages 1 and 3 were used for experiments. HUVECs were incubated for 4 h with 3 ng/mL of TNF-α. For the test, the labeled THP-1 cells were added to 4 × 10^5^ adherent TNF-α-treated HUVECs in a 24-well plate and incubated for 1 h, then the nonadherent cells were removed by two gentle washes with PBS and the number of bound monocytes counted by fluorescence microscopy.

### 2.7. Statistical Analysis

All data are expressed as the mean ± SEM. Differences in the mean values among different groups were analyzed by one-way ANOVA and a subsequent post hoc Dunnett test. A value of *P* < 0.05 was considered statistically significant.

## 3. Results

### 3.1. The Expression of Adiponectin Was Located in Macrophages of Atherosclerotic Lesions from Patients and Cholesterol-Fed Rabbits

**Figure 2:**
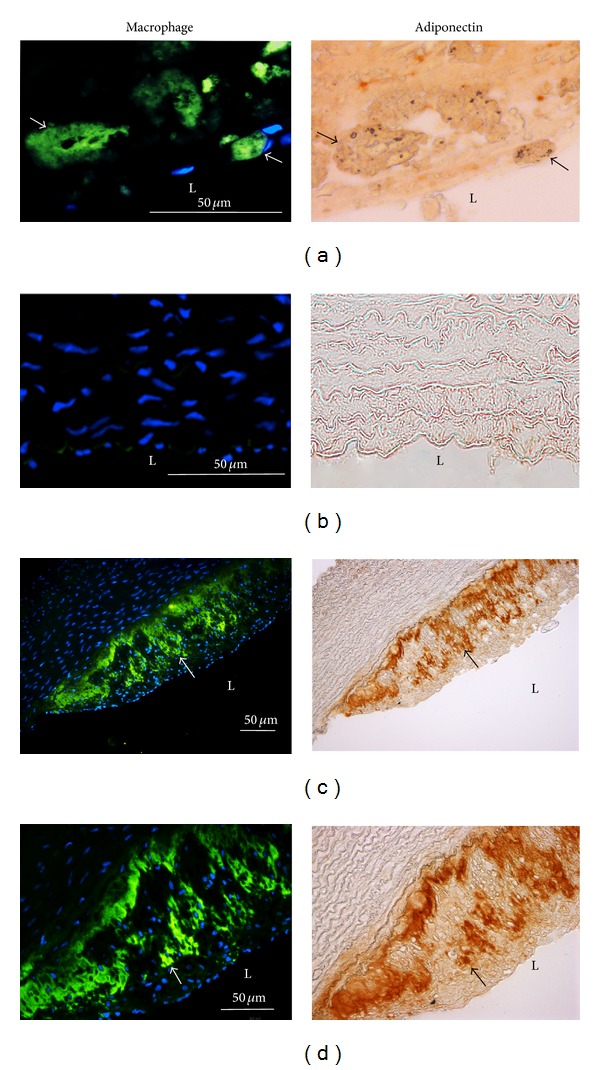
The expression of adiponectin was located in macrophages of atherosclerotic lesions from patients and cholesterol-fed rabbits by immunohistochemistry. Arterial serial sections from human atherosclerotic lesions (a), rabbits fed regular chow (b), or 2% cholesterol-containing diet for 6 weeks ((c), (d)) were stained for macrophages or adiponectin antibodies. Nuclei were stained by DAPI. L represents the vascular lumen. Bar = 50 *μ*m.

To investigate the adiponectin expression was associated with macrophages* in vivo*, the atherosclerotic lesions of human artery and cholesterol-fed rabbits were used and immunohistochemical staining was performed to detect the adiponectin expression. Adiponectin expression was observed mainly in atherosclerotic lesions of human patients, especially in the presence of macrophages, identified using antibody against macrophages (Figure 2(a)). As shown in Figure 2(b), weak adiponectin staining was seen in the normal group, while the cholesterol-fed group showed strong adiponectin staining in macrophages (Figure 2(c)). As shown in higher magnification, all of the adiponectin staining was present in macrophages (Figure 2(d)). Results of immunohistochemistry indicate that adiponectin expression was closely associated with macrophages.

### 3.2. TG and Δ2TG Enhanced Adiponectin mRNA and Protein Expression in THP-1 Cells

**Figure 3:**
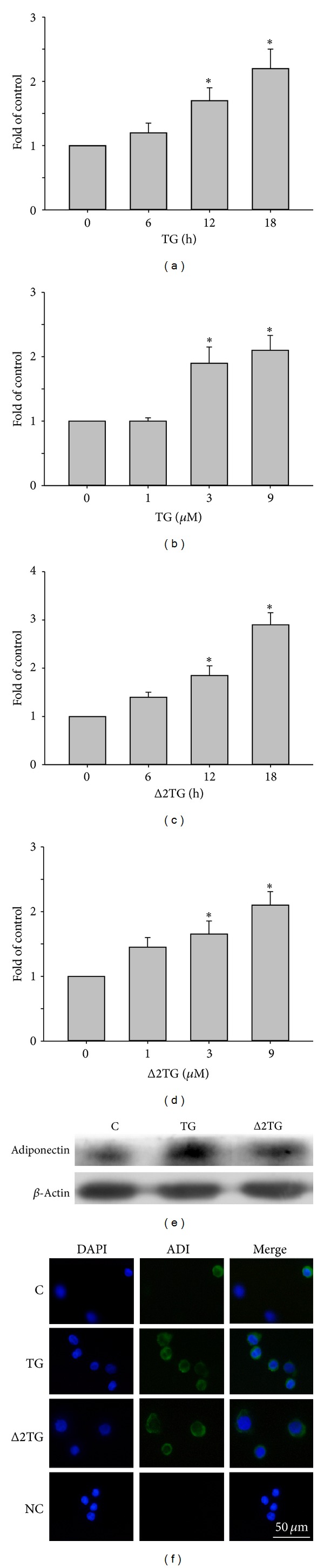
Troglitazone (TG) and Δ2troglitazone (Δ2TG) enhanced adiponectin mRNA and protein expression in THP-1 cells. ((a)–(d)) The expression of adiponectin mRNA was examined by quantitative RT-PCR. Macrophages were treated with 9 *μ*M of TG for the indicated time (a) or with the indicated concentration of TG for 18 h (b). In addition, macrophages were treated with 9 *μ*M of Δ2TG for the indicated time (c) or with the indicated concentration of Δ2TG for 18 h (d). GAPDH was used as the internal control. (e) Macrophages were incubated for 18 h with 9 *μ*M of TG or Δ2TG and adiponectin protein expression was measured in cell lysates by Western blotting. *β*-actin was used as the loading control. (f) Macrophages were treated for 18 h with 9 *μ*M TG or Δ2TG, and then, the distribution of adiponectin was analyzed by immunofluorescent microscopy. The merged images of adiponectin staining and DAPI were shown on the right panel. Adiponectin expression is indicated by green fluorescence (FITC) and nuclei by blue fluorescence (DAPI). The level of adiponectin expression was higher in TG or Δ2TG-treated cells. Scale bar = 50 *μ*m. *P < 0.05 as compared to the untreated cells.

When the cytotoxicity of TG or Δ2TG for THP-1 cells was detected by the MTT assay after 24 h of incubation, cell viability was not affected by the presence of 1–9 *μ*M of TG or Δ2TG (data no shown). To determine the optimal conditions for TG or Δ2TG-induced adiponectin mRNA expression by THP-1 cells, we first performed time-response and dose-response studies in which THP-1 cells were cultured with various concentrations of TG or Δ2TG for various time intervals. Adiponectin mRNA expression was induced in a time-dependent manner after treatment with 9 *μ*M of TG for 6, 12, or 18 h (1.2 ± 0.1, 1.8 ± 0.2, and 2.6 ± 0.4, resp., of control levels) (Figure 3(a)). The induction caused by the two highest time course being significant. Adiponectin mRNA expression was induced in a dose-dependent manner after treatment with 1, 3, or 9 *μ*M of TG for 18 h (1.0 ± 0.0, 1.9 ± 0.3, and 2.0 ± 0.3, resp., of control levels) (Figure 3(b)). The induction caused by the two highest concentrations was being significant. Δ2TG also enhanced adiponectin mRNA expression in THP-1 cells in both time- (Figure 3(c), 1.5 ± 0.1, 2.0 ± 0.2, and 3.0 ± 0.2, resp., of control levels) and dose-dependent manners (Figure 3(d), 1.4 ± 0.2, 1.7 ± 0.2, and 2.2 ± 0.2, resp., of control levels). To illustrate the expression and cellular localization of the* de novo* synthesized adiponectin protein in macrophages with TG or Δ2TG treatment was also studied by Western blotting and immunofluorescence staining. THP-1 cells were incubated with or without 9 *μ*M TG or Δ2TG for 18 h; then Western blotting was performed. TG or Δ2TG treatment resulted in a significant increase in adiponectin expression (Figure 3(e)). As shown in Figure 3(f), adiponectin expression was weak in untreated cells (C), while THP-1 cells treated with 9 *μ*M of TG or Δ2TG for 18 h showed strong adiponectin expression in the cytoplasm. In all subsequent experiments, unless otherwise specified, 9 *μ*M TG or Δ2TG were used.

### 3.3. TG Induced Adiponectin mRNA Expression through a PPAR*γ*-Dependent Pathway Whereas Δ2TG Enhanced Adiponectin mRNA Expression through a PPAR*γ*-Independent Pathway in THP-1 Cells

**Figure 4:**
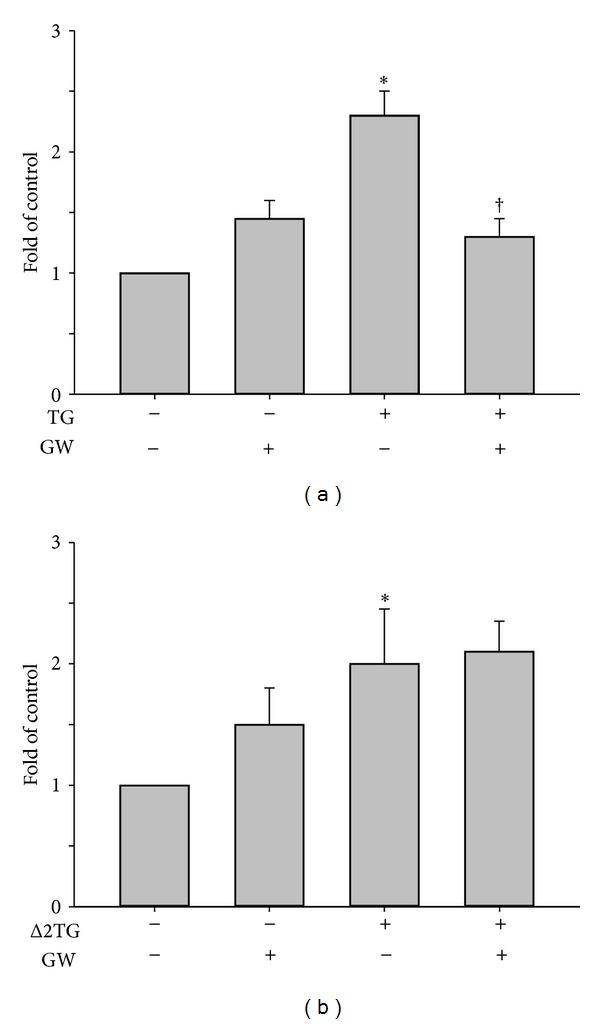
PPAR*γ* antagonist GW9662 abolished the TG-stimulated adiponectin mRNA expression and had no effect on Δ2TG-enhanced adiponectin mRNA expression in THP-1 cells. Macrophages were incubated for 1 h with 5 *μ*M GW9662 (a PPAR*γ* inhibitor) and then for 18 h with or without 9 *μ*M TG (a) or Δ2TG (b) in the continued presence of the inhibitor, and then, adiponectin mRNA expression was measured by quantitative RT-PCR. *P < 0.05 as compared to the untreated cells. ^†^ *P* < 0.05 as compared to the TG or Δ2TG-treated cells, respectively.

PPAR*γ* has emerged as a key regulator of adipocyte and macrophage function. PPAR*γ* activation is closely associated with potential effects on the expression and secretion of adiponectin [8]. To examine whether the effect of TG or Δ2TG on adiponectin mRNA expression is dependent on PPAR*γ*, we employed a PPAR*γ* antagonist, GW9662, and abolished TG-induced adiponectin mRNA expression (Figure 4(a)). In contrast, it had no effect on the upregulated adiponectin mRNA expression by Δ2TG treatment (Figure 4(b)). These data suggested that TG induced adiponectin mRNA expression through a PPAR*γ*-dependent pathway whereas Δ2TG enhanced adiponectin mRNA expression through a PPAR*γ*-independent pathway in THP-1 cells.

### 3.4. Both TG and Δ2TG Enhanced Adiponectin mRNA Expression in THP-1 Cells through AMPK Activation

**Figure 5:**
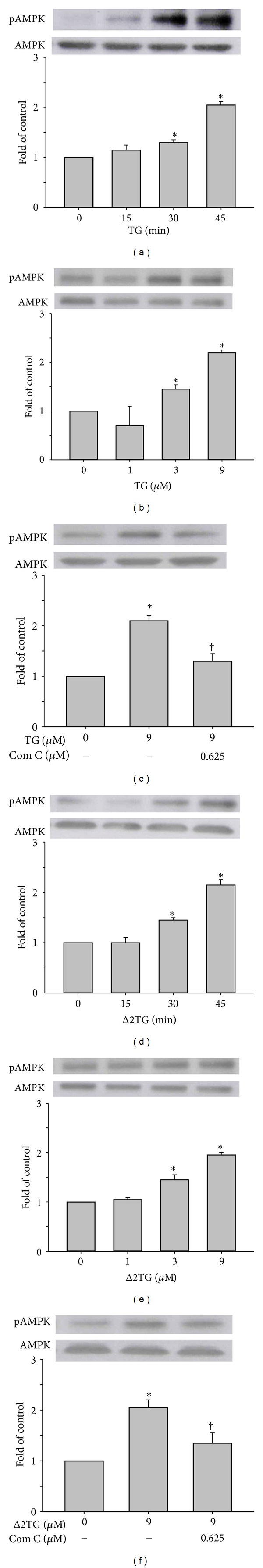
TG and Δ2TG enhanced AMPK phosphorylation. Macrophages were treated with 9 *μ*M of TG or Δ2TG for the indicated time ((a), (d)) or with the indicated concentration of TG or Δ2TG for 45 min ((b), (e)). ((c), (e)) Macrophages were incubated for 1 h with compound C (an AMPK inhibitor) and then for 45 min with or without 9 *μ*M TG or Δ2TG in the continued presence of the inhibitor, and then, the phosphorylated AMPK expression was measured in cell lysates by Western blotting. AMPK was used as the loading control. *P < 0.05 as compared to the untreated cells. ^†^ *P* < 0.05 as compared to the TG or Δ2TG-treated cells.

**Figure 6:**
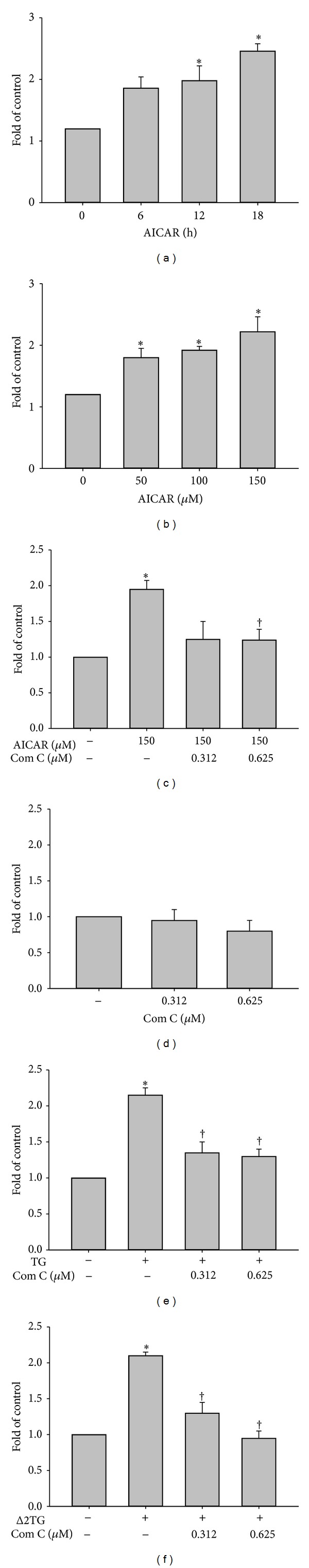
TG and Δ2TG enhanced adiponectin mRNA expression was mediated through the AMPK pathway in THP-1 cells. The expression of adiponectin mRNA was examined by quantitative RT-PCR. Macrophages were treated with 150 *μ*M of AICAR (an AMPK activator) for the indicated time (a) or with the indicated concentration for 18 h (b). Macrophages were treated with compound C (an AMPK inhibitor) for the indicated concentration and then with (c) or without (d) AICAR for 18 h and then adiponectin mRNA expression was measured by real-time PCR. Macrophages were incubated for 1 h with compound C and then for 18 h with or without 9 *μ*M TG (e) or Δ2TG (f) in the continued presence of the inhibitor, and then, adiponectin mRNA expression was measured by real-time PCR. *P < 0.05 as compared to the untreated cells. ^†^ *P* < 0.05 as compared to the TG or Δ2TG-treated cells.

Thiazolidinediones could activate AMPK in adipocytes, a pathway that increases fat oxidation and glucose transport [17]. THP-1 cells incubated with TG for 15, 30, or 45 min demonstrated a time-dependent increase in the phosphorylation of AMPK. The significant increase in phosphorylation was 1.3 ± 0.1-fold and 2.1 ± 0.1-fold at 30 min and 45 min treatment, respectively (Figure 5(a)). THP-1 cells incubated with TG for 1, 3, or 9 *μ*M for 45 min showed a dose-dependent increase in the phosphorylation of AMPK. The significant increase in phosphorylation was 1.4 ± 0.1-fold and 2.2 ± 0.1-fold at 3 *μ*M and 9 *μ*M treatment, respectively (Figure 5(b)). Cells treated with Δ2TG, paralleled to the result of TG treatment, showed the increase in AMPK phosphorylation in both time- (Figure 5(d), 1.0 ± 0.1, 1.4 ± 0.1, and 2.1 ± 0.1, resp., of control levels) and dose-dependent manners (Figure 5(e), 1.0 ± 0.1, 1.5 ± 0.1, and 2.0 ± 0.1, resp., of control levels). The phosphorylation of AMPK by both TG and Δ2TG could be abolished by compound C, an AMPK inhibitor (Figures 5(c) and 5(f)). To examine whether the upregulated effect of both TG and Δ2TG on adiponectin mRNA expression in THP-1 cells is through AMPK activation, AICAR, an AMPK activator was employed. AICAR treatment enhanced adiponectin mRNA expression in THP-1 cells in both time- and dose-dependent manners (Figures 6(a) and 6(b)). Compound C, an AMPK inhibitor, decreased the effect of AICAR on adiponectin mRNA expression (Figure 6(c)). Compound C treatment also decreased the upregulated effect of TG or Δ2TG on adiponectin mRNA expression (Figures 6(e) and 6(f)). These results TG- or Δ2TG-increased adiponectin mRNA expression was mediated through the AMPK phosphorylation.

### 3.5. TG and Δ2TG Decreased the Adhesion of THP-1 Cells to TNF-α-Treated HUVECs

**Figure 7:**
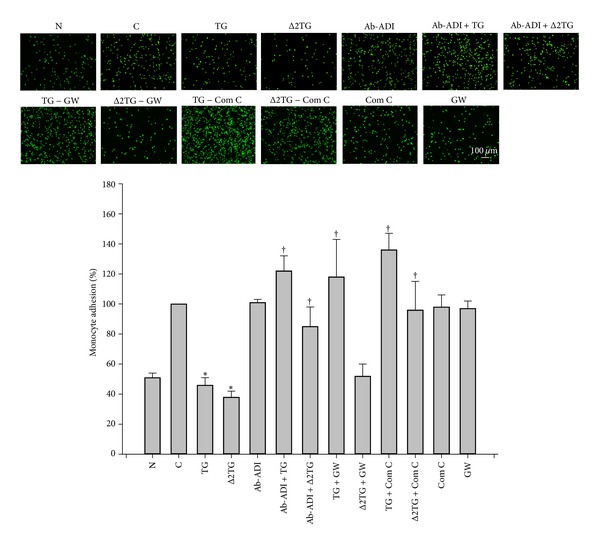
TG and Δ2TG reduced the adhesion of THP-1 cells to TNF-α-treated HUVECs. HUVECs were pretreated for 4 h with 3 ng/mL of TNF-α. THP-1 cells were left untreated or were pretreated for 1 h with 0.2 *μ*g/mL of purified antiadiponectin antibody (Ab-ADI) and then with 9 *μ*M TG or with Δ2TG for 18 h. In addition, THP-1 cells were left untreated or were pretreated for 1 h with 5 *μ*M GW9662 (GW) or 0.625 *μ*M compound C (Com C) and then with 9 *μ*M TG or with Δ2TG for 18 h in the continued presence of the inhibitor. The BCECF/AM-labeled THP-1 cells were added to TNF-α-treated HUVECs in a 24-well plate and incubated for 1 h, and then the nonadherent cells were removed by two gentle washes with PBS and the number of bound monocytes counted by fluorescence microscopy. N represents HUVECs without any treatment. C represents HUVECs with TNF-α treatment. *P < 0.05 as compared to the C cells. ^†^ *P* < 0.05 as compared to TG-treated cells and Δ2TG-treated cells, respectively. Bar = 100 *μ*m.

To explore the effects of TG and Δ2TG on the endothelial cell-leukocyte interaction, the adhesion of THP-1 cells to TNF-α-treated HUVECs was employed. As shown in the Figure 7(a), confluent HUVECs without any treatment (N) incubated with THP-1 cells for 1 h showed minimal binding, but adhesion was significantly increased when the HUVECs were pretreated with 3 ng/mL of TNF-α for 4 h (C). This effect was significantly decreased by treatment of THP-1 cells with 9 *μ*M TG or Δ2TG for 18 h. To assess the involvement of adiponectin in the TG or Δ2TG-reduced the number of THP-1 cells bound to TNF-α-treated HUVECs, the THP-1 cells was pretreated with antiadiponectin antibody. As shown in the Figure 7, when THP-1 cells were pretreated with 0.2 *μ*g/mL antiadiponectin antibody for 1 h, then incubated with either TG or Δ2TG for 18 h, the binding of THP-1 cells to TNF-α-treated HUVECs was significantly higher than that to non-antibody-treated THP-1 cells, showing that adiponectin plays an important role in the adhesion of THP-1 cells to TNF-α-treated HUVECs. Furthermore, GW9662 pretreatment attenuated TG-induced the inhibition of macrophages to TNF-α-treated HUVECs. In contrast, it had no effect on the inhibition of the adhesion of macrophages to TNF-α-treated HUVECs by Δ2TG treatment. TG- and Δ2TG-induced suppression on monocyte adhesion was inhibited by a selective AMPK inhibitor compound C. Taken together, these data indicate that the TG or Δ2TG-mediated inhibition on monocyte adhesion to TNF-α-treated HUVECs is, at least in part, mediated by the* de novo* synthesized adiponectin in THP-1 cells and the AMPK pathway.

## 4. Discussion

In this study, we demonstrated for the first time that TG and Δ2TG effectively increased adiponectin mRNA expression in a dose- and time-dependent manner in THP-1 cells. TG and Δ2TG also upregulate the adiponectin protein expression. Moreover,* de novo* synthesized adiponectin in macrophages significantly reduced monocyte adhesion to TNF-α-treated HUVECs via the AMPK pathway.

Adiponectin predominately secreted from adipose tissue, exerts multiple protective properties against obesity, diabetes, inflammation, cardiovascular diseases, and so on [18, 19]. Adiponectin is also detectable in several cell types, including endothelial cells, stellate cells and macrophages [4]. The present study demonstrated that adiponectin was significantly expressed in macrophages in atherosclerotic lesions of cholesterol-fed rabbits and humans during the development of cardiovascular diseases. Adiponectin was accumulated more preferably to the injured vascular wall than intact vessels. The previous study showed that the function of adiponectin expression in macrophage foam cells can significantly decrease triglyceride and cholesterol accumulation in these cells by reducing oxLDL uptake into the cells while enhancing HDL-mediated cholesterol efflux [20]. The treatment of macrophages with recombinant adiponectin protein lead to a reduction of reactive oxygen species and switched toward an anti-inflammatory phenotype [21]. Some insights have also been gained through work that overexpression of the adiponectin gene protected apoE-deficient mice from atherosclerosis by reducing lesion formation in the aortic sinus [22]. These results suggest that adiponectin expression in atherosclerotic lesions may play an important role in lipid metabolism and cholesterol efflux by modulating lipid metabolic signaling pathways for suppressing macrophage-to-foam cells transformation. All these investigations point to the anti-inflammatory and antiatherogenic role of adiponectin during atherosclerosis. Based on these findings, the regimen to increase adiponectin will provide a novel therapeutic strategy for cardiovascular and other related disorders.

Certain members of the thiazolidinediones family of the peroxisome proliferator-activated receptor (PPAR*γ*) agonists, such as TG and ciglitazone, possess a beneficial action against ROS, inflammation, and adipocytokine dysregulation [23, 24]. Moreover, thiazolidinediones-mediated PPAR*γ* activation has been shown to promote the differentiation of preadipocytes by mimicking certain genomic effects of insulin on adipocytes and to modulate the expression of adiponectin and a host of endocrine regulators in adipocytes [25]. 3T3-L1 adipocytes treated with TG upregulated adiponectin mRNA expression [26]. The present study demonstrated that TG and Δ2TG enhanced adiponectin mRNA and protein expression in THP-1 cells by quantitative real-time PCR, Western blot, and immunocytochemistry. Furthermore, GW9662, a PPAR-*γ* antagonist, treated macrophage was found to significantly decrease the TG-induced adiponectin mRNA expression while did not affect Δ2TG-induced adiponectin mRNA expression. The data suggest that TG strongly enhanced adiponectin expression in THP-1 cells through a PPAR-*γ*-signaling pathway, whereas Δ2TG did not. These findings indicate that the mechanism of the induction of adiponectin mRNA expression between TG and Δ2TG treatment was different. The previous report indicated that the structure of Δ2TG has the introduction of a double bond adjacent to the thiazolidinedione ring to abolish the ability of the resulting molecule to activate PPAR*γ* [27]. Δ2TG, a PPAR*γ*-inactive analogue of TG, was modestly more potent than their parent compounds in suppressing cell proliferation in cancer cells [28]. Because TG has some side effects [18], Δ2TG may be used as the additional alternative medications. However, additional studies are required to determine the affectivity and safety of Δ2TG for the prevention and treatment of cardiovascular disorders and inflammation.

AMPK, a fuel-sensing enzyme, which has been implicated in the regulation of glucose and lipid homeostasis and insulin sensitivity could perhaps account for the observed effects of thiazolidinediones on macrophages [29, 30]. AMPK is expressed in multiple tissues and is activated by diverse stimuli that increase the AMP-to-ATP ratio (e.g., exercise and hypoxia) as well as by hormones (e.g., adiponectin and leptin). Also, rosiglitazone has been shown to acutely activate AMPK in H-2Kb muscle cells, and when administered over a period of weeks they increase AMPK phosphorylation and activity in the liver and adipose tissue of rats [31]. TG can rapidly stimulate AMPK activity in isolated mammalian skeletal muscle [32]. Since the previous study had shown the ability of adiponectin to activate AMPK in myocytes and hepatocytes [33], we explored the effect of AMPK phosphorylation on adiponectin expression in TG or ΔTG-treated macrophages. Cells treated with TG or with Δ2TG showed the increase of AMPK phosphorylation in both time and dose-dependent manners. We also found that AICAR, an AMPK activator, enhanced the adiponectin mRNA expression in a time- and dose-dependent manner. In contrast, compound C, an AMPK inhibitor, decreased the upregulated effect of TG or Δ2TG on adiponectin mRNA expression. These results suggested that TG- or Δ2TG-increased adiponectin mRNA expression was mediated via the AMPK signaling pathway. A putative PPAR*γ* obligatory binding (PPAR-responsive element) site, C/EBPα, sterol-regulatory-element-binding proteins (SREBPs), and cAMP response element binding protein (CREB) were present in human and mouse adiponectin promoters, and point mutations at this site may lead to change TZD-induced adiponectin promoter transactivation [15]. The previous study reported that rosiglitazone promoted the modulation of AMPK-dependent CRTC2 (cAMP-dependent induct of the CREB regulated transcription coactivator 2) activity to influence hepatic gluconeogenesis [34]. Telmisartan, an angiotensin II type 1 receptor (AT_1_) blocker, can increase adiponectin production in white adipose tissue via a PPAR*γ*-independent mechanism, including the activation of AMPK-Sirt1 pathway [35]. Precise understanding of this molecular mechanism of AMPK activation involved in the Δ2TG-increased adiponectin mRNA expression will require further investigation.

Monocyte adhesion to endothelial surface has been considered as the major early step in the initiation of atherosclerosis and inflammation [36]. The earlier study demonstrated that the addition of recombinant adiponectin proteins had significantly inhibitory effects on monocyte adhesion and adhesion molecule expression in TNF-α-treated endothelial cells [37]. It has also been reported that adiponectin may inhibit both the inflammatory process and atherosclerosis by suppressing the migration of monocytes/macrophages and their transformation into macrophage foam cells in the vascular wall [5, 6]. In the present study, TG and Δ2TG reduced monocyte-EC adhesion under the inflammatory condition and this effect was mediated through the increase in adiponectin expression. The effects were blocked by the antiadiponectin antibody. The result demonstrated that the monocyte adhesion was reduced dependently by adiponectin expression. These inhibitory effects of monocyte adhesion were also abolished in the presence of an AMPK inhibitor, compound C. Consistent with the previous study, AMPK phosphorylation was involved in the inhibition of monocyte adhesion [38]. The present study demonstrated that the inhibitory effect of TG and Δ2TG on monocyte adhesion to TNF-α-treated HUVECs was mediated via* de novo* adiponectin expression and activation of AMPK signaling. On the basis of the probable involvement of adiponectin in monocyte recruitment to early atherosclerotic lesions, our findings suggest an additional mechanism by which TG and Δ2TG treatment may be important in preventing the progress of inflammation and atherosclerosis.

In conclusion, this study documented for the first time that TG and Δ2TG can upregulate the expression and function of adiponectin in human monocytes/macrophages. Furthermore, the upregulated expression of adiponectin by TG and Δ2TG inhibits monocyte adhesion to TNF-α-treated endothelial cells via activation of AMPK signaling pathway.

## References

[1] Mantovani A, Sica A, Locati M (2005). Macrophage polarization comes of age. *Immunity*.

[2] Auwerx J (1991). The human leukemia cell line, THP-1: a multifacetted model for the study of monocyte-macrophage differentiation. *Experientia*.

[3] Furukawa K, Hori M, Ouchi N (2004). Adiponectin down-regulates acyl-coenzyme A: cholesterol acyltransferase-1 in cultured human monocyte-derived macrophages. *Biochemical and Biophysical Research Communications*.

[4] Wolf AM, Wolf D, Avila MA (2006). Up-regulation of the anti-inflammatory adipokine adiponectin in acute liver failure in mice. *Journal of Hepatology*.

[5] Ouchi N, Kihara S, Arita Y (2001). Adipocyte-derived plasma protein, adiponectin, suppresses lipid accumulation and class A scavenger receptor expression in human monocyte-derived macrophages. *Circulation*.

[6] Tian L, Luo N, Klein RL, Chung BH, Garvey WT, Fu Y (2009). Adiponectin reduces lipid accumulation in macrophage foam cells. *Atherosclerosis*.

[7] Yki-Järvinen H (2004). Thiazolidinediones. *The New England Journal of Medicine*.

[8] Sharma AM, Staels B (2007). Review: Peroxisome proliferator-activated receptor *γ* and adipose tissue-understanding obesity-related changes in regulation of lipid and glucose metabolism. *Journal of Clinical Endocrinology and Metabolism*.

[9] Jung Y, Song S, Choi C (2008). Peroxisome proliferator activated receptor *γ* agonists suppress TNFα-induced ICAM-1 expression by endothelial cells in a manner potentially dependent on inhibition of reactive oxygen species. *Immunology Letters*.

[10] Ricote M, Huang JT, Welch JS, Glass CK (1999). The peroxisome proliferator-activated receptor*γ* (PPAR*γ*) as a regulator of monocyte/macrophage function. *Journal of Leukocyte Biology*.

[11] Tsai J-S, Chen C-Y, Chen Y-L, Chuang L-M (2010). Rosiglitazone inhibits monocyte/macrophage adhesion through de novo adiponectin production in human monocytes. *Journal of Cellular Biochemistry*.

[12] Parker JC (2002). Troglitazone: the discovery and development of a novel therapy for the treatment of Type 2 diabetes mellitus. *Advanced Drug Delivery Reviews*.

[13] Tsuchida A, Yamauchi T, Kadowaki T (2005). Nuclear receptors as targets for drug development: molecular mechanisms for regulation of obesity and insulin resistance by peroxisome proliferator-activated receptor *γ* CREB-binding protein, and adiponectin. *Journal of Pharmacological Sciences*.

[14] Whitehead JP, Richards AA, Hickman IJ, Macdonald GA, Prins JB (2006). Adiponectin—a key adipokine in the metabolic syndrome. *Diabetes, Obesity and Metabolism*.

[15] Shehzad A, Iqbal W, Shehzad O, Lee YS (2012). Adiponectin: regulation of its production and its role in human diseases. *Hormones*.

[16] Liang C-J, Wang S-H, Chen Y-H (2011). Viscolin reduces VCAM-1 expression in TNF-α-treated endothelial cells via the JNK/NF-*κ*B and ROS pathway. *Free Radical Biology and Medicine*.

[17] Greenberg AS, Obin MS (2006). Obesity and the role of adipose tissue in inflammation and metabolism. *The American Journal of Clinical Nutrition*.

[18] Fu Y (2014). Adiponectin signaling and metabolic syndrome. *Progress in Molecular Biology and Translational Science*.

[19] Guerre-Millo M (2008). Adiponectin: an update. *Diabetes and Metabolism*.

[20] Luo N, Liu J, Chung BH (2010). Macrophage adiponectin expression improves insulin sensitivity and protects against inflammation and atherosclerosis. *Diabetes*.

[21] Ohashi K, Parker JL, Ouchi N (2010). Adiponectin promotes macrophage polarization toward an anti-inflammatory phenotype. *Journal of Biological Chemistry*.

[22] Okamoto Y, Kihara S, Ouchi N (2002). Adiponectin reduces atherosclerosis in apolipoprotein E-deficient mice. *Circulation*.

[23] Hotamisligil GS (2006). Inflammation and metabolic disorders. *Nature*.

[24] Shoelson SE, Lee J, Goldfine AB (2006). Inflammation and insulin resistance. *Journal of Clinical Investigation*.

[25] Popovich DG, Li L, Zhang W (2010). Bitter melon (*Momordica charantia*) triterpenoid extract reduces preadipocyte viability, lipid accumulation and adiponectin expression in 3T3-L1 cells. *Food and Chemical Toxicology*.

[26] Maeda H, Saito S, Nakamura N (2013). Paprika pigments attenuate obesity-induced inflammation in 3t3-l1 adipocytes. *ISRN Inflammation*.

[27] Shiau C-W, Yang C-C, Kulp SK, Chen K-F, Chen C-S, Huang J-W (2005). Thiazolidenediones mediate apoptosis in prostate cancer cells in part through inhibition of Bcl-xL/Bcl-2 functions independently of PPAR*γ*. *Cancer Research*.

[28] Wei S, Yang J, Lee S-L, Kulp SK, Chen C-S (2009). PPAR*γ*-independent antitumor effects of thiazolidinediones. *Cancer Letters*.

[29] Iglesias MA, Ye J-M, Frangioudakis G (2002). AICAR administration causes an apparent enhancement of muscle and liver insulin action in insulin-resistant high-fat-fed rats. *Diabetes*.

[30] Sell H, Dietze-Schroeder D, Eckardt K, Eckel J (2006). Cytokine secretion by human adipocytes is differentially regulated by adiponectin, AICAR, and troglitazone. *Biochemical and Biophysical Research Communications*.

[31] Fryer LGD, Parbu-Patel A, Carling D (2002). The anti-diabetic drugs rosiglitazone and metformin stimulate AMP-activated protein kinase through distinct signaling pathways. *The Journal of Biological Chemistry*.

[32] LeBrasseur NK, Kelly M, Tsao T-S (2006). Thiazolidinediones can rapidly activate AMP-activated protein kinase in mammalian tissues. *The American Journal of Physiology: Endocrinology and Metabolism*.

[33] Yamauchi T, Kamon J, Minokoshi Y (2002). Adiponectin stimulates glucose utilization and fatty-acid oxidation by activating AMP-activated protein kinase. *Nature Medicine*.

[34] Yoon Y-S, Ryu D, Lee M-W, Hong S, Koo S-H (2009). Adiponectin and thiazolidinedione targets CRTC2 to regulate hepatic gluconeogenesis. *Experimental and Molecular Medicine*.

[35] Shiota A, Shimabukuro M, Fukuda D (2012). Activation of AMPK-Sirt1 pathway by telmisartan in white adipose tissue: a possible link to anti-metabolic effects. *European Journal of Pharmacology*.

[36] Vogl-Willis CA, Edwards IJ (2004). High glucose-induced alterations in subendothelial matrix perlecan leads to increased monocyte binding. *Arteriosclerosis, Thrombosis, and Vascular Biology*.

[37] Ouchi N, Kihara S, Funahashi T, Matsuzawa Y, Walsh K (2003). Obesity, adiponectin and vascular inflammatory disease. *Current Opinion in Lipidology*.

[38] Chang M-Y, Huang D-Y, Ho F-M, Huang K-C, Lin W-W (2012). PKC-dependent human monocyte adhesion requires AMPK and Syk activation. *PLoS ONE*.

